# Synthesis Characterization and Physicochemical Properties of Rigid Alicyclic Polyimide Films Based on Bicyclo[2.2.2]oct-7-ene-2,3,5,6-tetracarboxylic Dianhydride

**DOI:** 10.3390/polym16223188

**Published:** 2024-11-16

**Authors:** José Manuel Pérez-Francisco, Carla Aguilar-Lugo, Larissa Alexandrova, María O. Gonzalez-Diaz, Rita Sulub-Sulub, María Isabel Loría-Bastarrachea, Manuel Aguilar-Vega

**Affiliations:** 1 Departamento de Ingeniería Química, Tecnológico Nacional de México/Instituto Tecnológico Superior de Coatzacoalcos, Carretera Antigua Minatitlán-Coatzacoalcos Km. 16.5, Coatzacoalcos 96536, Mexico; 2 Instituto de Investigación en Materiales, Universidad Nacional Autónoma de México, Ciudad Universitaria, Ciudad de México 04510, Mexico; carla.aguilar.lugo@gmail.com (C.A.-L.); laz@unam.mx (L.A.); 3 Conahcyt-Centro de Investigación Científica de Yucatán A.C., Mérida 97205, Mexico; maria.gonzalez@cicy.mx; 4 Laboratorio de Membranas, Unidad de Materiales, Centro de Investigación Científica de Yucatán A.C., Calle 43 #130 entre 32 y 34, Chuburna de Hidalgo, Mérida 97205, Mexico; rita.sulub@cicy.mx (R.S.-S.); marisa@cicy.mx (M.I.L.-B.)

**Keywords:** bicyclo[2.2.2]oct-7-ene-2,3,5,6-tetracarboxylic dianhydride, alicyclic polyimides, thermostable polyimides, membrane films gas separation

## Abstract

Four polyimides based on bicyclo[2.2.2]oct-7-ene-2,3,5,6-tetracarboxylic dianhydride (BTD), **BTD-MIMA, BTD-HFA, BTD-FND**, and **BTD-TPM**, with different rigid substituted diamines were synthesized. The chemical structure of the polyimides was corroborated by 1H NMR spectroscopy. These polyimides were soluble in organic solvents and presented molecular weights (Mn) between 39 and 70 KDa. **BTD-MIMA, BTD-HFA, BTD-FND**, and **BTD-TPM** showed thermal stability above 400 °C. These polyimides also presented high glass transition temperatures between 272 and 355 °C. The alicyclic moiety increased solubility compared with other rigid polyimides. Membrane films from **BTD-MIMA** and **BTD-HFA** exhibited the highest gas permeability compared to **BTD-FND** and **BTD-TPM**. The introduction of ortho-substituents in **BTD-MIMA** or bulky –CF3 groups in **BTD-HFA**, in combination with the alicyclic dianhydride fragment, prevented chain packing and enhanced macromolecular chain rigidity. In turn, there was a shift toward higher gas permeability coefficients for **BTD-MIMA** and **BTD-HFA**, with a moderate loss of CO_2_/N_2_ and CO_2_/CH_4_ selectivity, and they presented similar selectivities to those of other reported polyimides with alicyclic BTD moieties containing asymmetric fragments.

## 1. Introduction

Aromatic polyamides with high rigidity are appreciated because they present excellent thermal and chemical resistance with outstanding physical properties. However, they present some drawbacks, such as low solubility and poor processability, which restrict their widespread application [1,2]. Therefore, there have been studies focused on designing and synthesizing new processable polyimides with minimum effects on their thermal and chemical resistance and physicochemical properties [3]. A variety of structural modifications to the polyimide backbone, such as bulky substituents [4], noncoplanar monomers [1], and flexible and kinked units [5,6], can be employed to reduce interchain interactions and the stiffness of the polymer chain. These structural modifications can lower the melting temperature, improving solubility and processability. Previous studies have shown that polyimides derived from alicyclic monomers such as bicyclo[2.2.2]-oct-7-ene-2,3,5,6-tetracarboxilyc dianhydride or bicyclo[2.2.1]heptane tetracarboxylic dianhydride structures show high solubility in organic solvents and good thermomechanical and optical properties due to their rigid cyclic structure [7,8,9]. Recently, several polymers of intrinsic microporosity (PIMs) have been developed with a combination of alicyclic and Tröger’s base units that present higher solubility and stability for gas transport studies with high gas permeability coefficients and reasonable selectivity [10]. There are also reports of the gas transport properties of alicyclic polyimides derived from alicyclic dianhydrides with a dihydroxyl-functionalized spirobisindane [11]. There are reports of PIM polyimide copolymers containing alicyclic diamines combined with rigid dianhydrides [12]. These materials were developed while looking into the possibility of synthesizing tractable polyimides with minimum sacrifice of their excellent thermal and physicochemical properties and improved gas permeability and selectivity.

Here, we report the synthesis of four alicyclic polyimides, **BTD-MIMA**, **BTD-HFA**, **BTD-FND**, and **BTD-TPM**, derived from bicyclo[2.2.2]oct-7ene-2,3,5,6-tetracarboxilic dianhydride and four highly rigid diamines, 4,4′-methylenebis(2-isopropyl-6-methylaniline) [MIMA], 4,4′-(hexafluro-isopropylidene)dianiline [HFA], 4,4′-(9-fluorenylidene)dianiline [FND], and 4,4′-diaminotriphenylmethane [TMP]. The originality of this work lies in the unique combination of a rigid alicyclic aromatic fragment with rigid bulky pendant groups, which has not been extensively explored in the context of alicyclic polyimides. This approach is expected to result in enhanced chain stiffness, which not only improves the material’s physicochemical properties but also is expected to increase solubility and both gas permeability and selectivity for gas transport, as compared with other rigid planar polyimides derived from BTD tetracarboxilic dianhydrides. By demonstrating that these structural combinations can lead to better performance in membrane applications, the study provides new insight into the design of polyimides for optimized gas separation processes. The effects of structural differences, particularly bulkiness and rigidity, introduced by the diamine to the base alicyclic-tetracarboxilic unit due to lateral motifs, on the solubility, thermal resistance, and gas transport properties of these alicyclic polyimide membrane films are evaluated and discussed.

## 2. Experimental Materials

Bicyclo[2.2.2]oct-7ene-2,3,5,6-tetracarboxylic dianhydride [BTD], 4,4′-(9-fluorenylidene)dianiline [FND], nitrobenzene, pyridine [Py], benzoic acid, dimethyformamide [DMF], dimethylsulfoxide [DMSO], 1-methyl-2-pyrrolidone [NMP], ethanol, and lithium chloride [LiCl] were used as received from Sigma-Aldrich S.A. de C.V, Toluca, México. 4,4′-methylenebis(2-isopropyl-6-methylaniline) [MIMA] and 4,4′-(hexafluroisopropylidene)dianiline [HFA] were supplied by Acros Organics Co, (Antwerpen, Belgium). MIMA was purified three times by recrystallization from hexane, and HFA was purified by sublimation. 4,4′-diaminotriphenylmethane [TMP] was synthetized according to a procedure in the literature [13].

### 2.1. Polyimide Synthesis

**Scheme 1:**
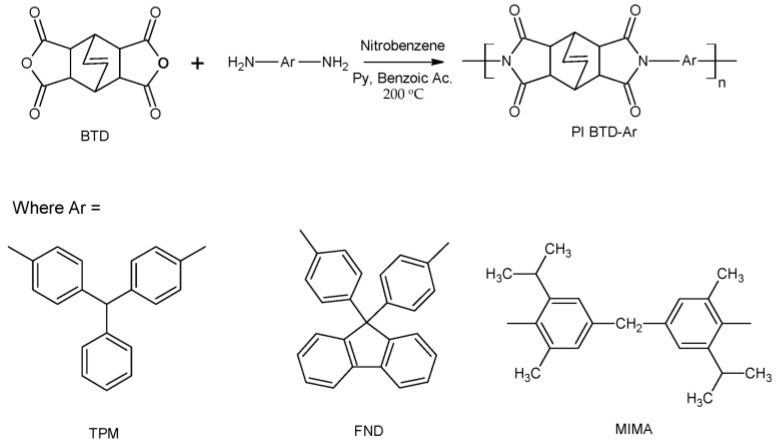
One-step synthesis of soluble BTD polyimides in nitrobenzene.

Preparation of the polyimides derived from BTD dianhydride and FND, MIMA, and TMP diamines was performed using a one-step method (see Scheme 1) [14]. In a typical polyimide synthesis, such as **BTD-MIMA**, a 50 mL three-necked flask equipped with a mechanical stirrer and nitrogen gas flow was charged with 2 mmol of MIMA and 5 mL of nitrobenzene. After the diamine was completely dissolved, 2 mmol of dianhydride BTD in 5 mL of nitrobenzene was added to the stirred solution, and the mixture was heated and kept at 80 °C for 1 h. Then, pyridine (4 mmol) was added and the solution was heated to 120 °C. After that, benzoic acid (4.0 mmol) was added, and the mixture was stirred at 150 °C for 3 h. Finally, the temperature was increased and kept at 200 °C for 24 h. The solution was poured into ethanol, and the obtained product was filtered and washed several times with ethanol. Finally, **BTDA-MIMA** was dried in a vacuum oven at 200 °C overnight. **BTD-TPM** and **BTD-FND** were synthesized according to the same procedure using the appropriate diamines, TMP and FDN.

**Scheme 2:**
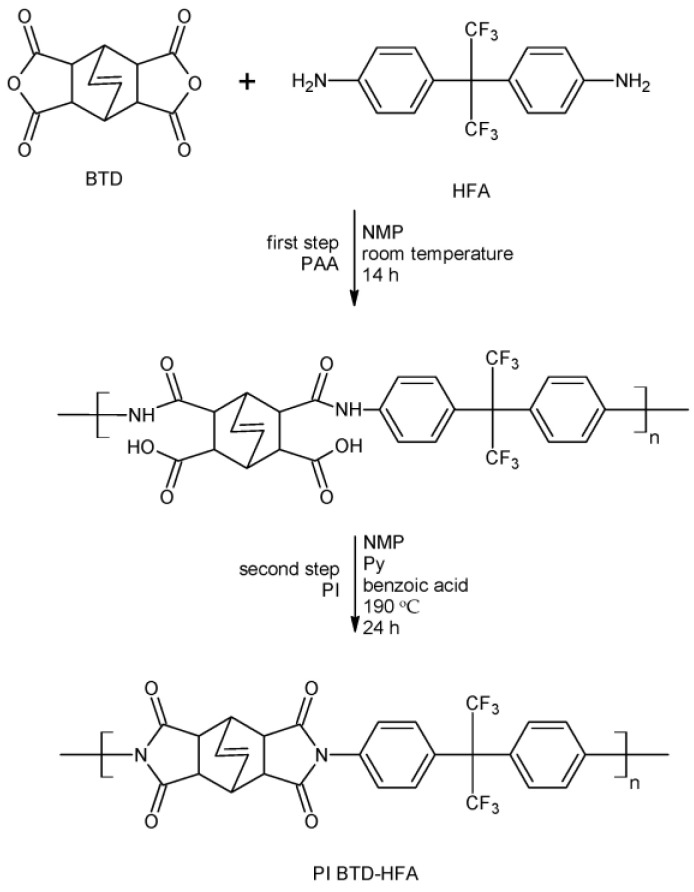
Two-step synthesis of BTD-HFA polyimide in NMP.

A two-step method was applied for the preparation of the polyimide derived from BTD dianhydride with HFA diamine (see Scheme 2). In the first step, a 50 mL three-necked flask equipped with nitrogen gas flow and a mechanical stirrer was charged with 2 mmol of HFA diamine and 5 mL of NMP. After the diamine was completely dissolved, 2 mmol of dianhydride BTD, 150 mg of LiCl, and 5 mL of NMP were added to the solution, and the mixture was stirred at room temperature for 14 h. For the second step, pyridine (4 mmol) was added, and the mixture was heated at 80 °C for 1 h. Then, the solution was heated slowly to 120 °C and benzoic acid (4.0 mmol) was added. The temperature was increased and kept at 190 °C for 24 h. The polymeric solution was poured into ethanol. The obtained polymer was washed several times with ethanol and dried in a vacuum oven at 200 °C overnight. This polymer was identified as **BTD-HFA**.

### 2.2. Membrane Preparation

Dense films of the polyimides were prepared using the solvent evaporation casting method. A 4% (*w/v*) polymer solution in NMP was prepared for **BTD-FND**, **BTD-MIMA**, or **BTD-TPM**, while for **BTD-HFA**, the same solution concentration was prepared in DMSO. The polymer solution was filtered and poured onto an aluminum plate surrounded by an aluminum ring. Subsequently, the solvent was evaporated slowly at 100 °C for 24 h. The obtained film was then removed and dried in a vacuum oven at 200 °C for 24 h to remove the residual solvent.

### 2.3. Characterization

^1^H NMR spectra were recorded on a Bruker Avance instrument at a proton frequency of 400 MHz using d_6_-DMSO as the solvent. The solubility of the polyimides was determined by dissolving 5 mg of polymer in 1 mL of solvent at room temperature for 24 h. The number-average molecular weight (*M*_n_), weight-average molecular weight (M_w_), polydispersity index (*M*_w_/*M*_n_), and intrinsic viscosity were determined by size exclusion chromatography (SEC) using a PerkinElmer Series 200 pump (Shelton, CT, USA), oven, and UV–Vis detector in parallel with a dual detector (Viscotek 270, Malvern, UK). The wavelength was set at 271 nm, the oven temperature was 70 °C, and the solvent flow rate was 0.3 mL/min. Polyethylene glycol standards were used as references. The samples were prepared by dissolving 1 mg of polymer in 1 mL of N,N-dimethylformamide (DMF). The software TotalChrom 6.3 from PerkinElmer (Shelton, CT, USA) was used for data acquisition and analysis. Two Polymer Labs Resipore (Agilent, Santa Clara, CA, USA) columns (3 μm, 300 mm × 4.6 mm, molecular weight range from 200 to 400,000) were used. The eluent was N,N-dimethylformamide containing 0.1% (*w/v*) LiBr. X-ray diffraction (XRD) measurements were performed with a Siemens 5000 X-ray (Bruker AXS LLC, Madison WI, USA) diffractometer using Cu-K_α_ radiation (λ = 1.54 Å) at 34 kV and 25 mA on polymer films. The measurements were made between 0 and 60° 2θ at a step rate of 0.04°. Thermogravimetric analysis (TGA) of BTD polyimide films was carried out using a thermogravimetric analyzer TGA-8000 (Perkin Elmer, Inc. Shelton, CT, USA) at a heating rate of 10 °C/min between 50 and 700 °C under a nitrogen atmosphere. The glass transition temperature (T_g_) was determined using a differential scanning calorimeter (DSC Q2000, TA Instruments, New Castel, DE, USA) at a heating rate of 10 °C/min under a nitrogen atmosphere in the range of 50–400 °C. The BTD polyimide film density was measured in a density gradient column (Techne Corp, Princeton, NJ, USA) based on aqueous calcium nitrate (Ca(NO_3_)_2_) solutions between 1.16 and 1.45 g cm^−3^ at 21 °C. The fractional free volume (*FFV*) was determined according to the following equation:

(1) $$FFV=\frac{V-V_{0}}{V}$$

where *V* is the specific volume, calculated from the measured density (*V* = 1/*ρ*); and *V_0_* is the volume occupied by polymeric chains, determined from equation *V*_0_ *= 1.3 V_w_*. The van der Waals volume (V_w_) was calculated using the Synthia module in Materials Studio v8.0 software.

### 2.4. Gas Transport Properties

The gas permeability coefficients (*P*) for the BTD polyimide membrane films were determined using a variable-pressure constant-volume method, as previously reported [15]. The pure gas permeability coefficients were measured at 35 °C and 2 atm upstream pressure for gases He, O_2_, N_2_, CH_4,_ and CO_2_. The purity of all gases used was >99.99% (Praxair Corp., Mérida, México) and *P* for each gas was calculated using the following equation:

(2) $$P_{A}=\frac{273.15}{76}\frac{Vl}{ATP_{0}}\left(\frac{dp}{dt}\right)$$

where *P_A_* is the permeability coefficient of gas A through the membrane, expressed in Barrer [1 Barrer = 1 × 10^−10^ cm^3^ (STP) cm cm^−2^ s^−1^ cmHg^−1^]; *V* is the constant volume of the permeation cell (cm^3^); *A* and *l* refer to the effective area (cm^2^) and the membrane thickness (cm), respectively; *T* is the temperature at which the measurement was carried out (K); *p_o_* is the pressure of the feed gas upstream; and (dp/dt) is the gas pressure increase with time under steady-state conditions measured in the permeation cell.

The apparent diffusion coefficient (*D*) was obtained using the time lag method with the following relationship:

(3) $$D=\frac{l^{2}}{6\theta }$$

where *l* is the membrane thickness and θ is the time lag. The apparent solubility coefficient (*S*) was subsequently calculated from ratio of the *P* and *D* coefficients, as follows:

(4) $$S=\frac{P}{D}$$

The ideal selectivity (*α*) of the membrane for gas A relative to gas B was defined as follows:

(5) $$\alpha =\frac{P_{A}}{P_{B}}$$

where *P_A_* and *P_B_* are the permeability coefficients for pure gas A and gas B, respectively.

## 3. Results and Discussion

### 3.1. Polyimide Synthesis and Characterization

The general reaction scheme leading to polyimide formation from diamines and dianhydrides consists of two discrete steps, polyacylation to give polyamic acid and imidization (cyclodehydration), which may be performed via two- or one-stage synthetic procedures [16,17]. Both of the above-mentioned reaction steps may be accelerated by benzoic acid [18]. In addition, the presence of tertiary amines also catalyzes the cyclodehydration step [A], which is why pyridine and benzoic acid were used in our syntheses. The single-stage high-temperature method is more convenient for the formation of polyimides than the traditional two-step method because it allows the formation of polymers with 100% imidization degree and minimum content of defect sites, such as isoimides, for example, in the polymer [B]. Under these reaction conditions, amic acid units are extremely short-lived intermediates and imidization occurs simultaneously with chain growth. Since nitrobenzene or m-cresol, the most widely used solvents in the single-stage method, are not as good for polyimides as dipolar aprotic solvents, only limited amounts of polyimides may be obtained using this method. In our case, **BTD-TPM**, **BTD-FND,** and **BTD-MIMA** polymers demonstrated good solubility in nitrobenzene at high temperature and, therefore, these polyimides were synthesized via the single-stage method, as shown in Scheme 1. Meanwhile, with **BTD-HFA** polyimide, we faced a gelation problem in nitrobenzene, and thus it was synthesized using a two-step method with NMP as the solvent, which is depicted in Scheme 2. The number-average molecular weight (M_n_), the weight-average molecular weight (M_w_), and the polydispersity index (PDI) of the polyimides are reported in Table 1. The obtained number-average molecular weights (*M_n_*) were between 39,000 and 70,000 Daltons, with PDI from 1.5 to 2.1. Considering the high temperatures and times employed, the molecular weight values were in the range expected for a polyimide polycondensation reaction. It is also important to point out that **BTD-HFA** had the lowest molecular weight among all of the prepared polyimides, but this was not a surprise since the single-stage method generally results in higher molecular weight polyimides than the two-stage one [19]. As for the polyimides obtained via the single-stage procedure, **BTD-FND** had a significantly lower molecular weight than the other two polymers, **BTD-MIMA** and **BTD-TMP**, probably due to its rigid fluorenyl cardo group. Polyimides derived from this diamine, but obtained via a two-stage procedure, are reported as very brittle films because of their low molecular weights [20]. The intrinsic viscosity values closely followed the trends observed in the *M_w_* and *M_n_* results.

**Figure 1:**
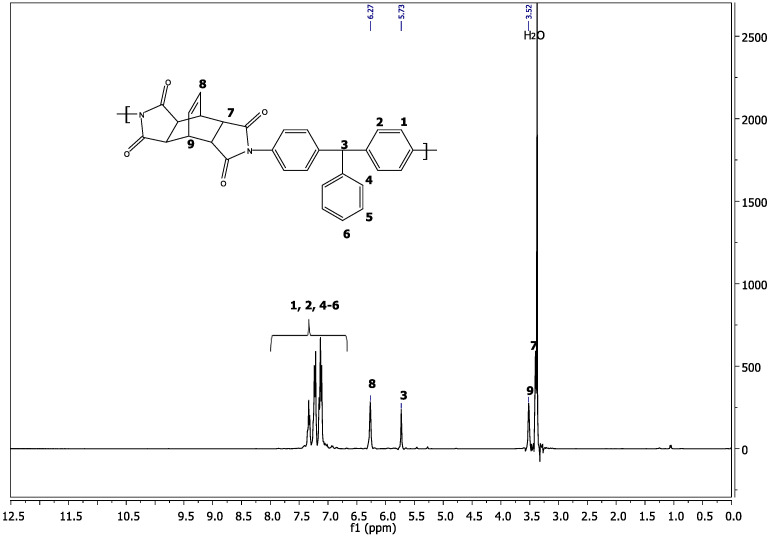
BTD-TPM ^1^H-NMR spectra.

The chemical structure of all BTD polyimides was confirmed by ^1^H-NMR spectroscopy. As an example, Figure 1 shows the peak assignments in the **BTD-TPM** ^1^H-NMR spectrum (^1^H-NMR spectra of **BTD-MIMA**, **BTD-FND,** and **BTD-HFA** are given in the Supplementary Materials, Figures S1–S3). All spectra exhibited three peaks around δ 3.60, 3.52, and 6.27 ppm, which were attributed to the protons on the bicyclic moiety, while the proton signals from the diamine fragment were influenced by their chemical environment. For example, for **BTD-TPM** (Figure 1), the peaks at δ = 7.11−7.33 ppm corresponded to protons on different aromatic rings, and the signal at δ = 5.73 ppm was assigned to the methylene protons in the TPM group. The spectrum of **BTD-HFA** displayed two doublets between 7.2 and 7.6 ppm, and the spectrum of **BTD-FND** showed signals between 7 and 9 ppm. In the case of **BTD-MIMA**, the signals at δ 7.19 and 7.01 ppm were assigned to the aromatic protons in the MIMA fragment, while the region between δ 2.6 and 1.01 ppm showed three additional signals at 2.50, 1.96, and 1.03 ppm, corresponding to the protons of the methyl and isopropyl ortho-substituents. Moreover, no presence of N-H, δ around 10 to 12 ppm, was found in **BTD-HFA,** which was an indication that the polyamic acid cyclization to polyimide was complete.

The solubility of the resulting polyimides was evaluated in several solvents. As can be seen in Table 2, **BTD-MIMA**, **BTD-FND,** and **BTD-TPM** bicyclic polyimides were highly soluble at room temperature in aprotic solvents such as DMSO, NMP, DMF, and DMAc. **BTD-MIMA** was the only one soluble in CHCl_3_, while **BTD-FND** and **BTD-TPM** were the only ones soluble in TCE (1,1,2,2-tetrachloroethane). On the other hand, **BTD-HFA** was soluble in DMSO and partially soluble in DMF. All polyimides were observed to be insoluble in 1,2-dichloroethane (DCE) and tetrahydrofuran (THF). The results indicated that the aliphatic substitution on the phenyl rings in MIMA allowed a higher solubility in different solvents. The alicyclic polyimide obtained from condensation with HFA diamine was only soluble in DMSO, which seemed to be related to the –CF3 substitution symmetry. These results reflected that the diamine used, particularly one with large bulky aliphatic substituents, also had a great effect on the solubility of the polyimides; the introduction of bulky and side groups resulted in excellent solubility, as reported in the literature [21,22].

### 3.2. Thermal Analysis

TGA and DSC measurements were used to evaluate the thermal degradation (*T_d_*) and glass transition temperature (*T_g_*) of **BTD-MIMA**, **BTD-FND, BTD-TPM**, and **BTD-HFA**, as shown in Table 3. No melting peaks were detected by DSC for the as-synthesized BTD polyimides, only their glass transition, suggesting that all BTD polyimides studied here were amorphous. The amorphous nature of **BTD-MIMA**, **BTD-FND, BTD-TPM**, and **BTD-HFA** was confirmed by X-ray diffraction (*DRX*) measurements. The glass transition temperatures (*T_g_*) reported for these polyimides were in the range of 272 to 355 °C. However, the experimental *T_g_* of **BTD-FND** was not possible to determine since, as was previously reported [7], it is >350 °C, which is close to the onset of the decomposition temperature. Among the polyimides characterized, **BTD-HFA** showed the highest *T_g_* value due to the presence of bulky –CF_3_ groups, which increased the rotational barriers and inhibited local segmental motions, imparting a higher rigidity to this polyimide [7,23].

**Figure 2:**
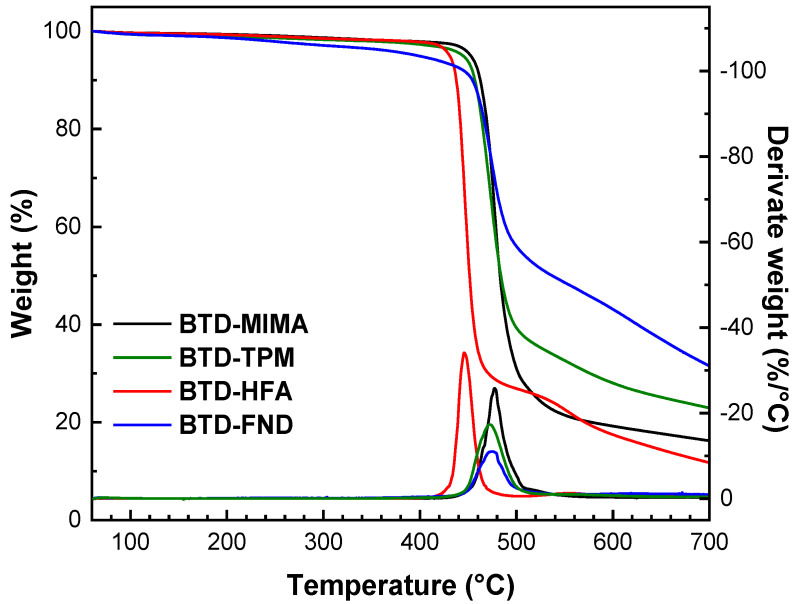
TGA thermograms of the BTD-based polyimides.

The onset of the decomposition temperature (*T_d_*) for the BTD-based polyimides is reported in Table 3, while the TGA thermograms are shown in Figure 2. As noted, the *T_d_* for the BTD polyimides was found between 437 and 460 °C under a nitrogen atmosphere. For all BTD polyimides, the TGA thermograms showed only one degradation step, which was attributed to main chain decomposition, except for **BTD-HFA**, which had a small shoulder at 540 °C. It can be seen that **BTD-HFA**, which possesses –CF_3_ groups in its structure, exhibited the lowest thermal stability of the four materials, presenting a *T_d_* of 437 °C and the highest weight loss at 700 °C. **BTD-HFA** also presented the lowest molecular weight of all of the samples, which may have affected its thermal resistance, while **BTD-MIMA** had the highest *T_d_* and it presented the highest molecular weight (see Table 1). By comparing the thermal decomposition temperatures of the synthesized BTD polyimides with similar aromatic polyimides reported by other researchers [2,4,6,14,24,25], the alicyclic polyimides from BTD presented T_d_ values close to those of fully aromatic polyimides but slightly lower due to the aliphatic segment of their alicyclic structure.

The BTD polyimide density increased in the order **BTD-MIMA** < **BTD-TPM** < **BTD-FND** < **BTD-HFA**. The largest density corresponded to **BTD-HFA** due to the presence of bulky –CF_3_ groups in the main chain. On the other hand, the isopropyl pendant groups in **BTD-MIMA** tended to decrease the density due to lower packing between polymer chains. The latter was corroborated by a larger *FFV* for **BTD-MIMA**, as reported in Table 3, compared to **BTD-TPM** and **BTD-FND,** which was quite close to the one presented by **BTD-HFA**. Bulky –CF_3_ groups, as in **BTD-HFA,** in similar fluorinated polymers tend to present a larger *FFV* [15,26,27].

### 3.3. X-Ray Diffraction

**Figure 3:**
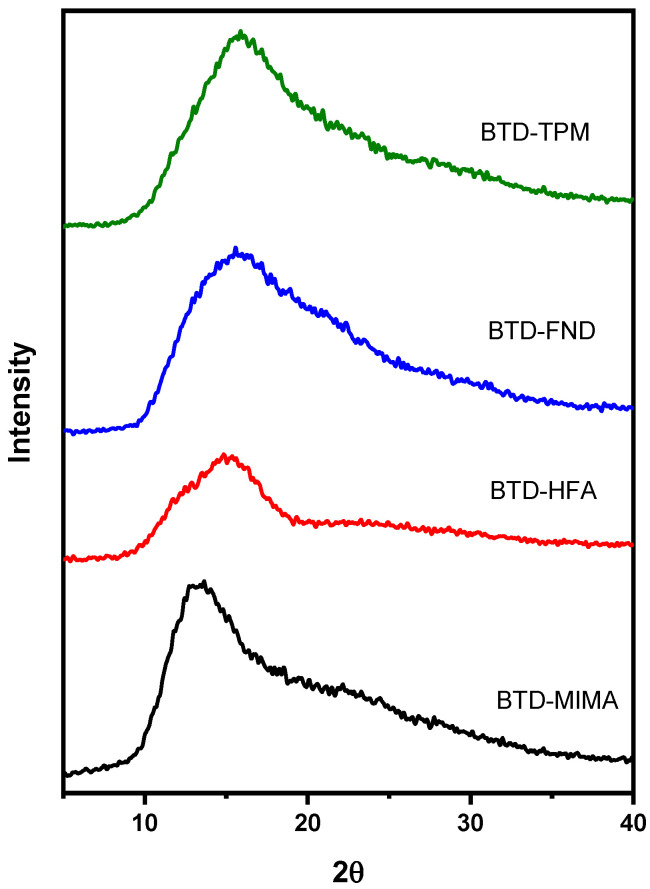
XRD patterns of BTD-based polyimides.

Figure 3 shows the *DRX* patterns for **BTD-TPM**, **BTD-FND**, **BTD-HFA**, and **BTD-MIMA**. All of them presented a wide amorphous halo indicative of the lack of crystallinity in the high rigidity polyimides. The maxima of the amorphous halos were used to calculate the *d*-space, which is related to the average distance between polymer chains. The *d*-space calculated using Bragg’s law $d=\frac{n\lambda }{2sin\theta }$ indicated that **BTD-TPM** and **BTD-FND** showed similar *d*-spaces at around 6.17 Å while the *d*-space in **BTD-HFA** increased to 6.58 Å. The largest *d*-space was found for **BTD-MIMA** at 7.35 Å. The increase in *d*-space was attributed to the bulky lateral substitutions from the main chain in the diamine used for polyimide synthesis, –CF_3_ in **BTD-HFA** and –C(CH_3_)_2_ in **BTD-MIMA** [28].

### 3.4. Gas Transport Through Rigid BTD-Based Polyimide Membranes

The gas permeability coefficients (*P*) at 2 atm upstream pressure and 35 °C measured using a constant-volume/variable-pressure method for **BTD-TPM**, **BTD-FND**, **BTD-HFA**, and **BTD-MIMA** polyimides are presented in Table 4. Polyimides **BTD-MIMA** and **BTD-HFA** had the highest permeability coefficients found in this work. The O_2_, N_2_, and CO_2_ permeability coefficients were similar for both polyimides. Different bulky backbone substituents in diamine fragments affect chain packing, which in turn affects the permeability coefficient. Santiago-García et al. [29] reported that *ortho*-substituents inhibit chain packing, resulting in higher gas permeability coefficients, as seen in the case of polyimide **BTD-MIMA**. Moreover, polymers containing –CF₃ groups, such as **BTD-HFA**, are well known to exhibit higher gas permeability compared to those without such substituents. The increased free volume facilitates the diffusion of gas molecules through the material [2,3,4,5,30,31]. On the other hand, the presence of pendant phenyl or cardo fluorenylidene groups on polyimides contributes to increased rigidity and improved packing density [30,32]. While this structural enhancement in **BTD-TPM** and **BTD- FND** led to more efficient gas transport, its impact was less pronounced compared to **BTD-MIMA** and **BTD-HFA**. The latter polyimides offered superior packing efficiency, resulting in enhanced performance in gas separation.

**BTD-FND** and **BTD-TPM** presented the lowest *FFV*; correspondingly, they presented lower permeability coefficient values. The pure gas permeability coefficients for all polyimides, except **BTD-MIMA**, decreased in the order P_He_ > P_CO2_ > P_O2_ > P_N2_ > P_CH4_, which was related to the kinetic diameter of the penetrant gases. **BTD-MIMA** followed the order P_CO2_ > P_He_ > P_O2_ > P_CH4_ > P_N2_, as has been reported in microporous polyimides [14], polymers with *ortho*-substituted diamines [29], and PIMs [33,34]. FFV and *d*-spacing provide valuable information that complements the understanding of gas transport behavior among different polymer membrane materials [35,36]. In this case, the *P* value increased in correlation with an increase in *d*-spacing. An increase in *d*-spacing indicates a less densely packed structure within the polymer, which can create larger pathways available for gas transport. This enhanced mobility can lead to higher permeability, as the free volume within the material allows for easier diffusion of gases. However, **BTD-MIMA** exhibited a slight decrease in FFV compared to **BTD-HFA**. This may have been attributed to the presence of methyl and isopropyl groups in the MIMA moiety, which do not enhance FFV but instead contribute to increased chain stiffness, resulting in improved gas permeability for **BTD-MIMA** [37].

Table 4 shows the ideal selectivity for BTD-based polyimide membranes for the gas pairs CO_2_/CH_4_ and CO_2_/N_2_. Polyimides **BTD-FND** and **BTD-HFA** exhibited the highest selectivity for all gas pairs reported, while highly permeable polyimide **BTD-MIMA** presented the lowest selectivity, which was consistent with the reported trade-off between permeability and selectivity commonly found for gas separation in polymeric membranes [24]. However, the gas selectivity was better for **BTD-HFA** than for **BTD-MIMA**, with little difference in CO_2_ permeability.

**Table 4:** Pure gas permeability coefficients and ideal selectivity for BTD-based rigid polyimides.

Polyimide	Permeability (Barrer)					Selectivity	
	He	O_2_	N_2_	CH_4_	CO_2_	α_CO2/CH4_	α_CO2/N2_
**BTD-MIMA**	165.9	42.8	10.7	13.9	224.6	16.1	20.8
**BTD-HFA**	274.6	41.9	9.9	7.1	212.9	29.9	21.4
**BTD-FND**	61.6	8.6	1.7	1.7	47.7	28.5	27.4
**BTD-TPM**	40.5	5.9	1.5	1.6	32.3	20.2	21.5
**PIM-EA-TB [38]**		2150	525	699	7140	10.2	13.6
**Ac-CoPI-TB1 [10]**	919	331	81	33	1366	17	16.8
**Ac-CoPI-TB2 [10]**	540	166	33	33	555	17	16.8
**BTA-CANAL2 [12]**		392	112	125	1995	16	17.8
**BTA-CANAL4 [12]**		115	26	30	600	20	23.1
**BTA-CANAL3 [12]**		40	9.6	11	230	22	23.9
**POBI-1 [39]**		57	13.7	13.5	334	24.7	24.3
**POBI-2 [39]**		61	14.9	14.6	356	24.4	23.8
**POBI-3 [39]**		39	6.6	6.2	250	40.3	37.8
**POBI-4 [39]**		41	7.1	6.7	267	39.9	37.6

The apparent gas diffusion (*D*) and gas solubility (*S*) coefficients are shown in Table 5 and Table 6, respectively. Also, the diffusion selectivity (α(D_A_/D_B_)) and solubility selectivity (α(S_A_/S_B_)) are included. Table 5 shows that the rigid alicyclic BTD polyimide membranes had the general trend *D*_O2_ > *D*_CO2_ > *D*_N2_ > *D*_CH4_ for the apparent diffusion coefficients. **BTD-MIMA** and **BTD-HFA** exhibited diffusion coefficients 4 to 10 times larger than **BTD-FND** and **BTD-TPM**, which could be correlated with the introduction of *ortho*-substituent groups and bulky –CF_3_ groups, which increased the diffusion coefficients as a result of higher average distance between polymer chains (*d*-spacing) and higher *FFV* [29,40]. In Table 6, the apparent solubility coefficients follow the order *S*_CO2_ > *S*_CH4_ > *S*_O2_ > *S*_N2_, making CO_2_ the most soluble of all of the gases tested and following closely with gas condensability. As in the case of the diffusion coefficients, an increase in *FFV* resulted in higher solubility coefficients.

**Figure 4:**
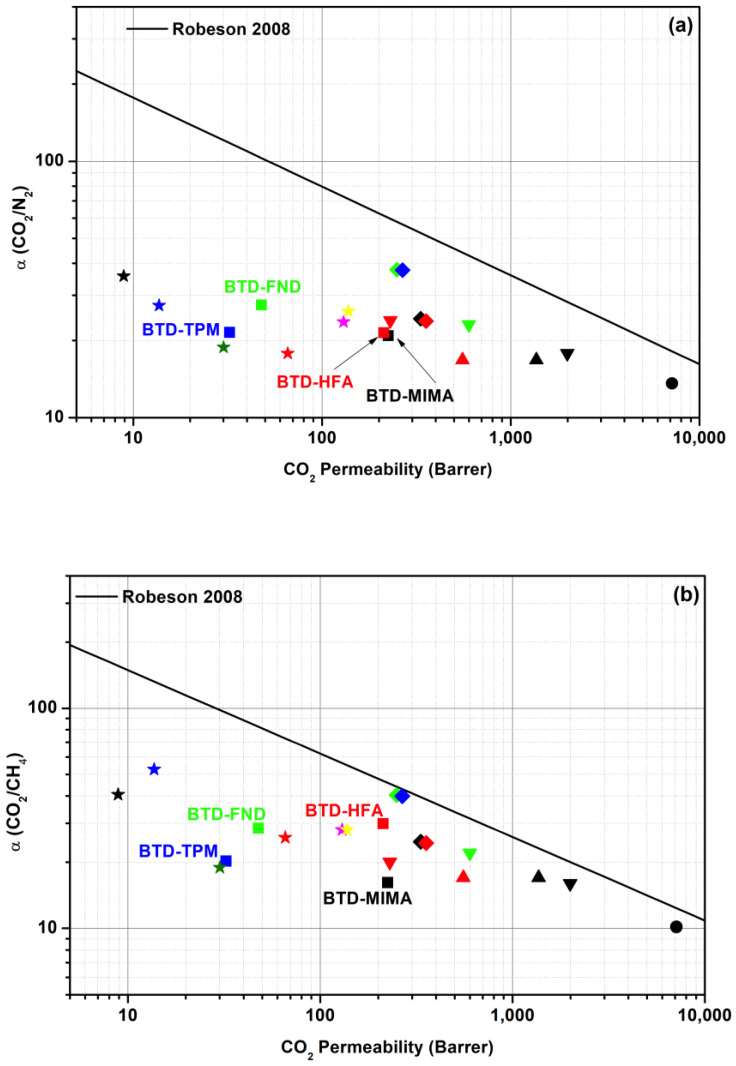
(**a**) CO_2_/N_2_ and (**b**) CO_2_/CH_4_ separation performance of BTD membranes (squares) compared to PIM-EA-TB (circle) [38], Ac-CoPI-TB series (triangles) [10], BTA-CANAL series (inverse triangles) [12], POBI-I series (diamonds), Matrimid (black star) [41], 6FDA-FSC (red star), BTDA-FSBC (green star), DSDA-DDBT (blue star) [40], BC-SBIDA (pink star), and BCBr4-SBIDA (yellow star) [11].

A comparison of the ideal selectivity of **BTD-TPM**, **BTD-FND**, **BTD-HFA**, and **BTD-MIMA** synthesized polyimides (gas permeability coefficient of the most permeable gas and ideal selectivity of a pair of gases CO_2_/N_2_ and CO_2_/CH_4_) with Matrimid^®^ and other recently published polyimides is shown in Figure 4 [11,12,38,39,40,41], where it can be observed that the correlation between permeability (*P*) and selectivity (α) for the gas pairs CO_2_/N_2_ and CO_2_/CH_4_ was related to the most permeable gas (CO_2_) in the form of a Robeson plot [24,40,42,43]. **BTD-HFA** and **BTD-MIMA** approached the upper boundary with larger CO_2_ permeability than Matrimid and similar CO_2_/N_2_ and CO_2_/CH_4_ selectivity closer to the upper boundary. The permeability relationship for **BTD-FND** and **BTD-TPM** showed low permeability without a considerable increase in selectivity. The **BTD-MIMA** and **BTD-HFA** performance could be attributed to the fact that polymers with *ortho*-substituted groups and bulky –CF_3_ groups, combined with a rigid backbone structure, presented high *FFV*, which was associated with higher pure gas permeability without selectivity loss. As a result, the gas permeability coefficients of **BTD-MIMA** and **BTD-HFA** increased more than 10 times compared to Matrimid^®^, while the gas pair selectivity decreased around 1–2 times [14,29,44]. This performance was similar to that reported by Mahmoud et al. [11], where they reported two alicyclic polyimides with ideal selectivity coefficients similar to the ones reported in the present work for **BTD-MIMA** and **BTD-HFA** and moderate permeability coefficients, which was the result of introducing alicyclic moieties and a spirobisindane diamine. **BTD-TPM**, **BTD-FND**, **BTD-HFA**, and **BTD-MIMA** presented lower permeability coefficients than PIM combined with a Tröger’s base polymer containing a similar alicyclic moiety, PIM-EA-TB [38], and also copolyimides with an alicyclic moiety and Tröger’s base combination [10], or rigid polyimides with two alicyclic moieties, one of them BTD, BTA-CANAL-2, BTA-CANAL-3, and BTA-CANAL-4 [12]. While the latter reported polymers all had higher CO2 permeability, they all presented lower CO_2_/N_2_ and CO_2_/CH_4_ selectivity, following the usual trade off. On the other hand, they presented similar CO_2_ permeability and selectivity values, which were quite close to the rigid polyimides prepared from spirobisbenzoxazole and 4,4′-(hexafluoroisopropylidene)diphthalic anhydride (6FDA), structures POBI-1 and POBI-2, or spirobisbenzoxazole and cyclohexanedianhydride (H’PMDA) polyimides POBI-3 and POBI-4 [39]. Overall, the permeability and selectivity behaviors of rigid **BTD-TPM**, **BTD-FND**, **BTD-HFA**, and **BTD-MIMA** bearing the alicyclic **BTD** fell between those of PIMs with a Tröger’s base containing a similar moiety and were similar to those of highly rigid spirobisbenzoxazoles.

## 4. Conclusions

Four polyimides based on bicyclo[2.2.2]oct-7ene-2,3,5,6-tetracarboxylic dianhydride (**BTD-MIMA**, **BTD-HFA**, **BTD-FND**, and **BTD-TPM**) with different rigid and *ortho*-substituted diamines were synthesized to test their solubility and processability. ^1^H NMR spectroscopy corroborated the structure of the four obtained alicyclic polyimides. The alicyclic polyamides presented high molecular weights (*Mn*) between 39 and 70 KDa. The four BTD-based polyimides showed high thermal stability with the onset of thermal decomposition above 430 °C. They also presented high glass transition temperatures between 272 and 355 °C, which compares well with BTDA and DSDA polyimides. The presence of the alicyclic moiety imparted good solubility in aprotic solvents and the solubility in chlorinated solvents was improved with the presence of bulky aliphatic *ortho*-substituents in the rigid diamine motif, **BTD-MIMA**. Furthermore, membranes from the four polyimides were prepared to test their potential for applications in gas separation. Membranes from **BTD-MIMA**, **BTD-HFA**, **BTD-FND**, and **BTD-TPM** exhibited high gas permeability, in particular **BTD-MIMA** and **BTD-HFA**, in comparison with commercial Matrimid and some other reported polyimides. The introduction of *ortho*-substituents in **BTD-MIMA** or bulky –CF_3_ groups in **BTD-HFA**, in combination with the alicyclic dianhydride fragment, prevented chain packing and enhanced macromolecular chain rigidity. In turn, there was a shift toward higher gas permeability coefficients with a moderate loss of CO_2_/N_2_ and CO_2_/CH_4_ selectivity compared to commercial Matrimid membranes and their homologous **BTD-FND** and **BTD-TPM** containing asymmetric diamine fragments.

## Figures and Tables

**Table 1:** Gel permeation chromatography analysis of bicyclic polyimides.

Polyimide	*M_w_* (Dalton)	*M_n_* (Dalton)	PDI	η ^a^ (dL/g)
**BTD-MIMA ^b^**	146,326	69,517	2.10	1.37
**BTD-HFA ^c^**	62,927	39,412	1.59	0.43
**BTD-FND ^b^**	68,052	43,419	1.56	0.48
**BTD-TPM ^b^**	99,611	48,303	2.06	0.63

^a^ Intrinsic viscosity (η) measured directly in the automatic viscometer of the SEC apparatus. ^b^ Obtained by single stage synthetic method. ^c^ Obtained by two stage synthetic method.

**Table 2:** Solubility of bicyclic polyimides at 25 °C.

Polyimide	NMP	DMF	DMAc	CHCl_3_	DCE	TCE	THF	DMSO
**BTD-MIMA**	+	+	+	+	−	−	−	+
**BTD-HFA**	−	±	−	−	−	−	−	+
**BTD-FND**	+	+	+	−	−	+	−	+
**BTD-TPM**	+	+	+	−	−	+	−	+
**DDE^1^**	+	+	+	−				+
**DDM^1^**	+	+	+	−	NR	NR	NR	+
**BAB^1^**	+	+	+	+	NR	NR	NR	+
**PI-DP2**	±	−	±	−	NR	NR	−	−
**PI-6F2**	+	+	+	+	NR	NR	+	+

**Table 3:** Thermal and physical properties of BTD-based polyimides.

Polyimide	*T_d_*(°C)	*T_g_*(°C)	*d*-Spacing (Å)	Density (g/cm^3^)	*V_w_* (cm^3^/mol)	*FFV*
**BTD-MIMA**	460	345	7.32	1.177	299.51	0.1232
**BTD-HFA**	437	355	6.56	1.427	256.57	0.1289
**BTD-FND**	450	-	6.23	1.289	296.01	0.1150
**BTD-TPM**	454	272	6.17	1.279	261.16	0.1070

**Table 5:** Apparent diffusion coefficients and diffusion selectivity of rigid BTD-based polyimides.

Polyimide	Diffusion Coefficient (10^−8^ cm^2^/s)				Diffusion Selectivity	
	O_2_	N_2_	CH_4_	CO_2_	α_CO2/CH4_	α_CO2/N2_
**BTD-MIMA**	31.4	7.3	2.9	9.5	3.3	1.3
**BTD-HFA**	32.0	6.6	2.4	10.7	4.4	1.6
**BTD-FND**	6.9	1.7	1.0	3.7	3.7	2.1
**BTD-TPM**	4.8	1.5	0.9	2.9	3.2	1.9

**Table 6:** Apparent solubility coefficients and solubility selectivity of rigid BTD-based polyimides.

Polyimide	Solubility Coefficient (10^−2^ cm^3^(STP)/cm^3^cm Hg)				Solubility Selectivity	
	O_2_	N_2_	CH_4_	CO_2_	α_CO2/CH4_	α_CO2/N2_
**BTD-MIMA**	1.36	1.47	4.72	23.47	4.97	15.96
**BTD-HFA**	1.31	1.49	2.96	19.90	6.72	13.35
**BTD-FND**	1.25	1.00	1.68	12.73	7.57	12.73
**BTD-TPM**	1.22	1.02	1.79	11.01	6.15	10.79

## Data Availability

The original contributions presented in the study are included in the article/Supplementary Material, further inquiries can be directed to the corresponding author.

## Supplementary Materials

The following supporting information can be downloaded at: https://www.mdpi.com/article/10.3390/polym16223188/s1, Figure S1. ^1^H-NMR spectrum of BTD-HFA; Figure S2. ^1^H-NMR spectrum of BTD-FND; Figure S3. ^1^H-NMR spectrum of BTD-MIMA.
