# Supplementary material for: Synthesis Characterization and Physicochemical Properties of Rigid Alicyclic Polyimide Films Based on Bicyclo[2.2.2]oct-7-ene-2,3,5,6-tetracarboxylic Dianhydride
Source: Polymers (Basel). 2024 Nov 16;16(22):3188. doi: 10.3390/polym16223188 (PMC11598778; doi:10.3390/polym16223188)
Supplement: Supplementary file 1 [file polymers-16-03188-s001.zip › polymers-3257250-supplementary.pdf]

Supplementary Material

Synthesis, characterization and gas transport properties of rigid alicyclic polyimides

José Manuel Pérez-Francisco[a], Carla Aguilar-Lugo[b], María O. Gonzalez-Diaz[c], Rita Sulub-Sulub[d], María Isabel Loría-Bastarrachea[d], Manuel Aguilar-Vega[*,d]

[a]Tecnológico Nacional de México/Instituto Tecnológico Superior de Coatzacoalcos, Carretera Antigua Minatitlán-Coatzacoalcos Km. 16.5, 96536, Coatzacoalcos, Veracruz, México

[b]Instituto de Investigación en Materiales, Universidad Nacional Autónoma de México, Ciudad Universitaria, Ciudad de México, 04510, México

[c]Conahcyt-Centro de Investigación Científica de Yucatán A.C., 97205 Mérida, Yucatán, México

[d]Laboratorio de Membranas, Unidad de Materiales, Centro de Investigación Científica de Yucatán A.C., Calle 43 #130 entre 32 y 34, Chuburna de Hidalgo, 97205, Mérida, Yucatán, México

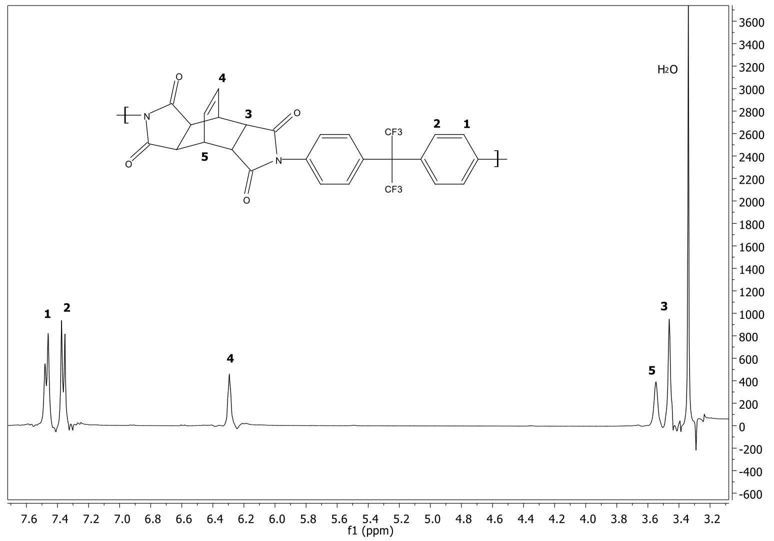

Figure S1. ^{1}H-NMR spectra of BTD-HFA

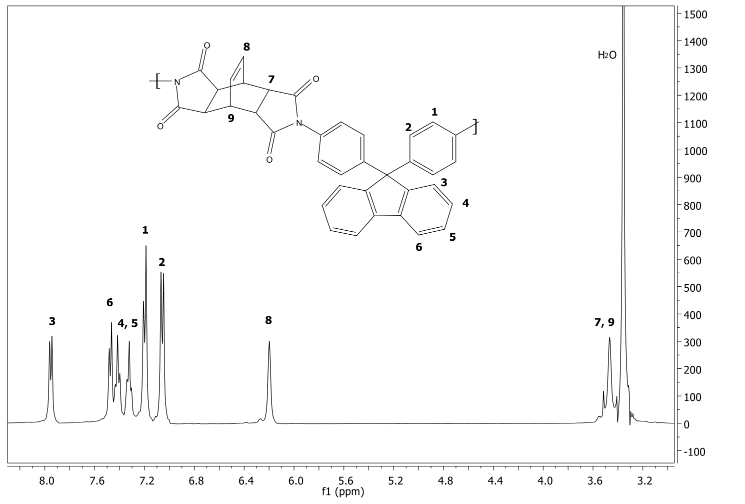

Figure S2. ^{1}H-NMR spectra of BTD-FND

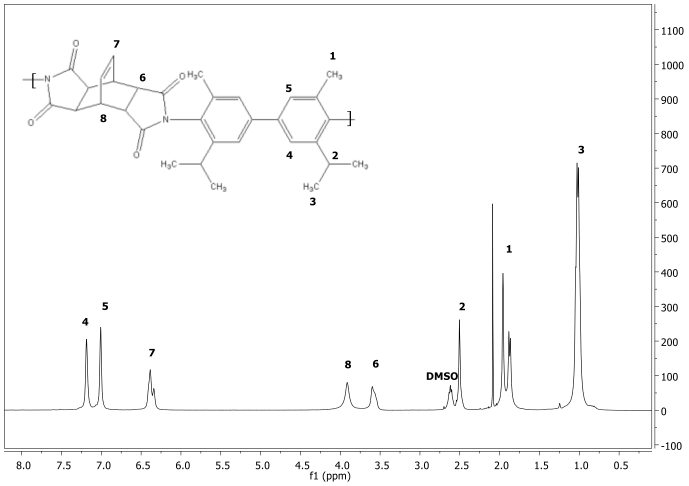

Figure S3. ^{1}H-NMR spectra of BTD-MIMA
